# Enhanced recovery in Cardiac surgery

**DOI:** 10.1186/1749-8090-10-S1-A75

**Published:** 2015-12-16

**Authors:** Gillian Hardman, Amal Bose, Helen Saunders, Antony H Walker

**Affiliations:** 1 Department of Cardiothoracic Surgery, Lancashire Cardiac Centre, UK

## Background/Introduction

Enhanced recovery after surgery (ERAS) is well established in other surgical specialties, accelerating recovery and improving outcomes.

We have developed and implemented an ERAS programme in our institution for patients undergoing first time, isolated coronary artery bypass grafts with adequate post-operative social support.

## Aims/Objectives

We report our early experience with enhanced recovery following cardiac surgery.

## Method

Thirty-seven patients were enrolled in the enhanced recovery programme between January and December 2014. These were propensity matched using surgeon, gender, status of operation (urgent/elective) number of coronary artery bypass grafts, EuroSCORE and logisticEuroSCORE. All patients were included in the retrospective analysis. Comparison between the groups was made using a t-test.

## Results

Mean post-operative length of stay was significantly reduced in the ERAS group, 4.05 (SD 1.43) days compared to 5.4 (SD 1.17) days in the non-ERAS group (p = 0.003). There were no hospital or Intensive Care Unit (ICU) readmissions in either group. Mean ICU length of stay was 1 night in both groups.

There was no mortality in either group. The rate of re-operation for bleeding was slightly increased in the ERAS group, although this was not significant. There was no significant difference in the frequency of use of endoscopic vein harvest between the two groups.

The proportion of cases carried out by non-consultant grade surgeons was higher in the ERAS group, although this did not reach statistical significance.

## Discussion/Conclusion

Following the successful implementation of an ERAS programme we demonstrate that enhanced recovery in Cardiac surgery is safe, with no increase in readmission or complication rates and a significantly reduced hospital length of stay.

